# Supplementary figures and images for: The COVID-19 pandemic and its impacts on diet quality and food prices in sub-Saharan Africa
Source: PLoS One. 2023 Jun 29;18(6):e0279610. doi: 10.1371/journal.pone.0279610 (PMC10309633; doi:10.1371/journal.pone.0279610)

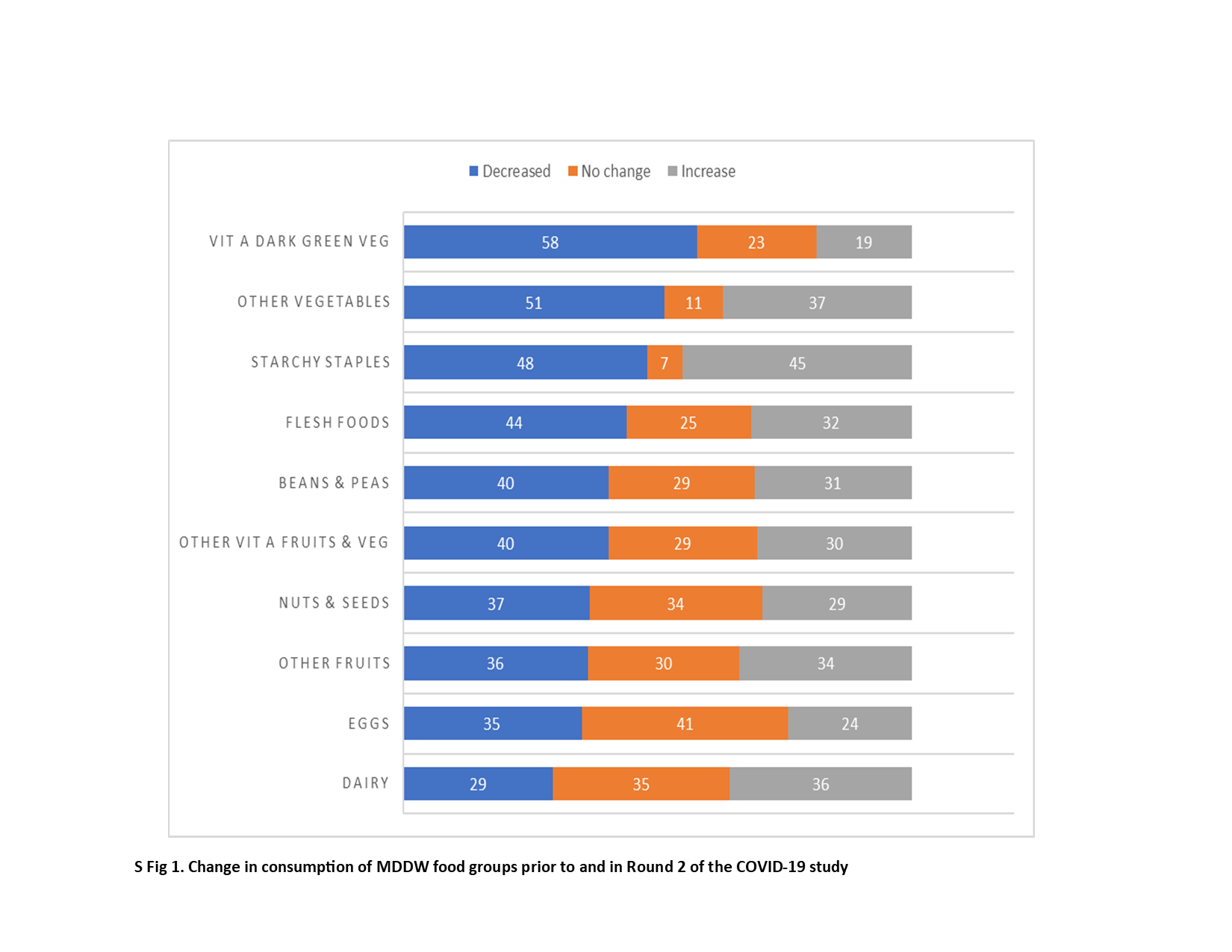

Supplement: S1 Fig — (TIF) [file pone.0279610.s001.tif]
